# ASSESSMENT OF PCXMC MONTE CARLO SIMULATIONS IN SLOT-SCANNING-BASED EXAMINATIONS: COMPARISON WITH IN-PHANTOM THERMOLUMINESCENT DOSIMETRY

**DOI:** 10.1093/rpd/ncac273

**Published:** 2022-12-29

**Authors:** A Piai, A Loria, P Tiberio, S Magnino, M Campoleoni, L M Sconfienza, A del Vecchio

**Affiliations:** Medical Physics Department, IRCCS Ospedale San Raffaele, 20132 Milan, Italy; Medical Physics Department, IRCCS Ospedale San Raffaele, 20132 Milan, Italy; Medical Physics Department, IRCCS Ospedale San Raffaele, 20132 Milan, Italy; Postgraduate School of Medical Physics, Università degli Studi di Milano, 20133 Milan, Italy; Medical Physics Unit, Foundation IRCCS Ca’ Granda Ospedale Maggiore Policlinico, 20122 Milan, Italy; Unit of Diagnostic and Interventional Radiology, IRCCS Istituto Ortopedico Galeazzi, 20161 Milan, Italy; Department of Biomedical Sciences for Health, Università degli Studi di Milano, 20123 Milan, Italy; Medical Physics Department, IRCCS Ospedale San Raffaele, 20132 Milan, Italy

## Abstract

Slot-scanning technology is nowadays a valid solution for the follow-up of chronic musculoskeletal disorders on children and adolescent patients, but there is no commercial software designed for simulating this X-ray beam geometry. PC Program for X-ray Monte Carlo (PCXMC) is a widespread Monte Carlo software developed for dose computation in projection radiography. In this study, experimental measurements were performed to evaluate its applicability in examinations with a slit-beam device. Physical phantoms corresponding to an adult and a 5-y-old child with calibrated thermoluminescent dosemeters were used for experiments. Different simulation approaches were investigated. Differences between measured and calculated organ doses ranged from −95 to 67% and were statistically significant for almost all organs. For both patients, PCXMC underestimated the effective dose of about 25%. This study suggests that PCXMC is not suited for organ dose evaluation in examinations with slot-scanning devices. It is still a useful tool for effective dose estimation when a proper correction factor is applied.

## Introduction

The amount of X-ray examinations has been increasing over the last decades, and nowadays X-ray diagnostics is a significant source of radiation exposure in general population. Presently, stochastic harm to humans from ionising radiation is assessed by equivalent doses in various organs or tissues in the body and by the effective dose^(1)^. However, the organ doses and the effective dose cannot be measured directly in patients undergoing X-rays examinations, so they are estimated with direct measurements in physical phantoms or with computational methods. Since experimental measurements are time-consuming and often complex to arrange, where possible calculations are the favored approach.

This study investigates the feasibility of PC Program for X-ray Monte Carlo (PCXMC)^(2)^ Monte Carlo (MC) software for computing organ doses and effective dose in examinations with a slit-beam imaging device. The use of slot-scanning technology allows acquiring true-to-size images of the whole body with a single acquisition, removing the need for digital stitching and avoiding the magnification errors because of the divergent X-ray beam. Among slit-beam digital radiography (DR) devices, the EOS system is nowadays the principal commercial solution^(3)^. EOS was developed for orthopedic imaging, and it has shown prominent results for the imaging of chronic skeletal and musculoskeletal conditions, especially for the evaluation of scoliosis, limb length discrepancy and posture complications^(4, 5)^. For these reasons, it is often used for follow-up purposes on children and adolescent patients, special practices that may require accurate dose evaluations for risk assessments. However, as far as we know, at present no commercial solution has been developed for dose estimations in such conditions.

Nowadays, all modern X-ray dosimetry methods in diagnostic radiology rely on MC calculations. Indeed, if sufficient data on the radiological technique are available, they provide reasonably accurate dose estimations without the need for time-consuming measurements with physical phantoms. The MC algorithms explicitly compute stochastic events, using random numbers for simulating the transport of radiation in the human body. Several MC implementations are available, such as ITS3^(6)^, EGS4^(7)^, EGS5^(8)^, GEANT3^(9)^, GEANT4^(10)^ and others. Among them, PCXMC is a widespread MC software developed for computation of equivalent and effective doses in projection radiography and fluoroscopy. The program allows the computation of organ doses for patients of different ages and sizes in freely adjustable X-ray projections and its accuracy for dose calculation in DR has been fully demonstrated^(11–13)^.

Although it was not developed for slot-scanning examinations, different authors applied PCXMC also for the simulation of EOS acquisitions^(5, 14–16)^. Two different approaches can be found in the literature: Law *et al.*^(14)^ divided the scan range into contiguous 0.5mm–high slot beams and performed several simulations across the whole spine, whereas Hui *et al.*^(15)^ performed only one simulation, selecting field dimension to include the full body. However, to the best of our knowledge, the use of PCXMC has never been formally validated with experimental measurements.

Thereby, this study evaluated the applicability of PCXMC in order to identify a method for dose assessments in EOS examinations. For this purpose, different simulation approaches were investigated and the calculated doses were compared with those measured with physical phantoms and thermoluminescent dosemeters (TLDs).

## Materials and methods

Examinations were performed with the EOS imaging device. EOS is a biplane slot-scanning system that allows the simultaneous acquisition of anteroposterior (AP) and latero-lateral (LL) images of the body. The two X-ray tubes are coupled with output collimators that generate thin fan-shaped beams, with fixed vertical dimension of 0.5 mm. These slit-beam X-ray sources are set at 90° and aligned with two micro-grid detectors. This double X-ray detection system is enabled to slide along a vertical stand, allowing two planar acquisitions in one mechanical motion. Different speeds of the scanning tube can be selected. As such, since the radiation dose is directly related to the scan time, the dose to the patient can be affected by the variation of the speed level (SL).

PCXMC was applied for dose estimations on both adult and pediatric patients. Two anthropomorphic phantoms were used for experimental measurements with this purpose: an adult Alderson Rando phantom^(17)^, which simulates a normal adult male (175 cm, 73.5 kg), and a 5-y-old CIRS Atom phantom (110 cm, 19 kg)^(18)^. The phantoms are transected horizontally into 35 and 19 25mm–thick slices, respectively, with section numbers starting from the top of the head down to the pelvic floor.

TLDs type GR-200A (LiF: Mg, Cu, P) were employed. Dosemeters were positioned in the most radiosensitive organs with guidance from human anatomy CT atlas. To allow the placement of the dosemeters into the phantoms, the holes of the selected positions were filled with specifically cut plugs. Table 1 lists, for each investigated organ, the number of employed TLDs and their positions inside the phantoms. To evaluate skin dose, TLDs were attached to the external phantom surface, anteriorly, posteriorly, and on the right and left sides. For the adult patient, breast attachments were used to estimate the dose to the breasts.

**Table 1:** Number of TLDs used for each organ dosimetry and their position inside the phantoms. The slice number followed by the number of dosemeters (in parenthesis) in that slice is reported in the ‘position’ columns.

	Adult patient		Pediatric patient	
Organ	N° TLD	Position	N° TLD	Position
Active bone marrow	4	33(2); 27(2)	11	26(2); 24(4); 11(3); 3(2)
Brain	7	3(1); 2(2); 1(4)	4	4(2); 3(2)
Breasts	4	Behind breast attachments	2	12
Colon	5	26(2); 25(2); 24(1)		
Heart	5	18(2); 17(2); 15(1)	3	14(1); 13(1); 12(1)
Kidneys			4	18(2); 17(2)
Liver	13	24(1); 23(2); 22(2); 21(3); 20(3)	7	17(4); 16(4)
Lungs	16	19(6); 18(6); 16(4)	6	14(3); 13(3)
Esophagus	2	16(1); 14(1)	2	12(1); 10(1)
Oral mucosa	2	7(1); 6(1)	1	6
Pancreas	3	23	2	18
Prostate	2	34(1); 33(1)	1	24
Skin	12	External surface	12	External surface
Small intestine	8	27(2); 26(2); 25(2); 24(2)	7	22(4); 19(3)
Spleen	5	23(2); 22(2); 21(1)		
Stomach	7	22(2); 21(2); 20(3)	4	17(2); 16(2)
Testicles	2	35	2	25
Thyroid	2	9	2	9
Urinary bladder	5	32(1); 31(4)	2	23
**Total**	**104**		**72**	

**Figure 1:**
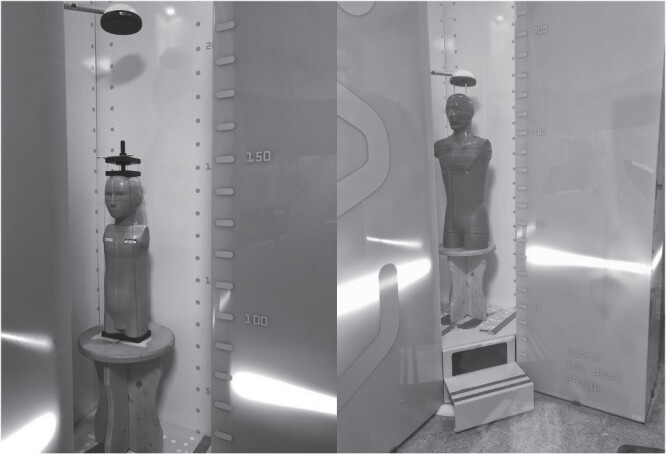
Acquisition setup. The pediatric (on the left) and adult (on the right) anthropomorphic phantoms were placed at the center of the EOS scanning chamber and centered using laser-lights guides.

The phantoms were placed in the center of the scanning chamber, as shown in Figure 1. In order to study the impact of the X-ray tube velocity, whole-body examinations at three SLs, corresponding to increasing delivered doses, were performed for the pediatric patient. For the adult phantom, only SL 4 was investigated, as the most used in the clinical practice. Details about the acquisition parameters are reported in Table 2. In order to accumulate a sufficient dose and to reduce the TLD statistical uncertainty, each acquisition was repeated three times.

**Table 2:** Acquisition parameters of AP and LL projections of pediatric and adult examinations.

	**Pediatric**						**Adult**	
	**SL 2: 15.2 cm/s**		**SL 3: 11.4 cm/s**		**SL 4: 7.6 cm/s**		**SL 4: 7.6 cm/s**	
**Parameter**	AP	LL	AP	LL	AP	LL	AP	LL
kV	83	102	83	102	83	102	90	105
mA	200	200	200	200	200	200	250	250
Area (cm^2^)	2912	2912	2912	2912	2912	2912	4200	3360
DAP (mGy cm^2^)	102.20	156.41	153.28	234.61	204.38	312.81	454.17	519.27

**Figure 2:**
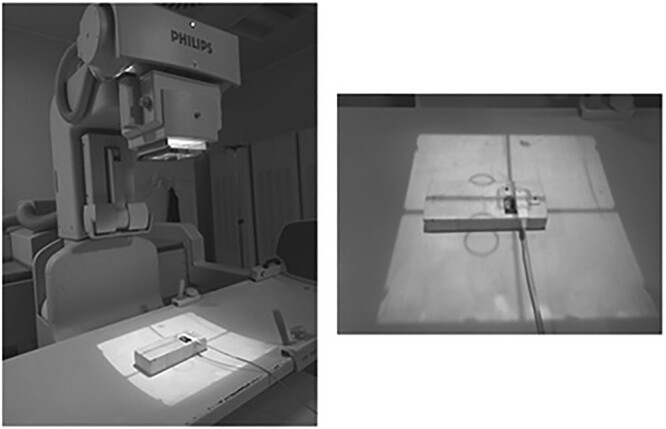
Picture of the calibration setup. TLD cards were place side-by-side with the RaySafe dosemeter on a Styrofoam support. Unfiltered cards were used for the measurements.

TLDs were calibrated in air with a DR device. Measurements were performed at a fixed source-to-detector distance of 100 cm with a RaySafe X2 dosemeter with calibration traceable to national standards. Calibration parameters were chosen in order to simulate the beam quality of the EOS system. To guarantee the same backscattering and exposure conditions, TLDs were placed side-by-side with the RaySafe dosemeter on a Styrofoam support, properly designed so that the chamber and TLD crystals were at the same distance from the source. Five measurements were performed, exposing groups of nine TLDs to each dose. A picture of the calibration setup is shown in Figure 2.

The same dosemeters were used in all the experimental measurements. Before each irradiation, dosemeters were pre-annealed to remove any residual signal. Furthermore, in order to avoid systematic errors, the position of each TLD inside the phantom was changed from one measurement to the other.

TLD responses were individually corrected for individual sensitivity and background radiation and doses were calculated applying the calibration curve. For each organ, the equivalent dose was calculated as the average of doses measured by TLDs placed inside the organ, and normalised to a single acquisition.

Effective dose was computed according to ICRP 103 recommendations^(1)^, as the sum

$$ \mathrm{D}={\sum}_{\mathrm{T}}{\mathrm{W}}_{\mathrm{T}}{\sum}_{\mathrm{R}}{\mathrm{W}}_{\mathrm{R}}{\mathrm{D}}_{\mathrm{T},\mathrm{R},} $$

where W_R_ is the radiation weighting factor (being unity for X-rays), D_T,R_ is the absorbed dose to an organ or tissue measured with TLDs and W_T_ is the tissue weighting factor. Doses to oral mucosa, small intestine and active bone marrow were assumed attributable to doses to salivary glands, colon and bone surface, respectively; dose to remainder tissues was computed as the average of doses to heart, kidneys (for pediatric), spleen (for adult), oral mucosa, pancreas, prostate and small intestine.

The combined uncertainty was calculated according to the Guide to the Expression of Uncertainty Measurements recommendations (uncorrelated input quantities)^(19)^. The sources of uncertainties are reported in Table 3.

**Table 3:** Uncertainty budget for organ dose estimation.

**Parameter**	**Source**	**Type**	**Calculation method**
CALIBRATION CURVE			
TLD counts	Repeatability	A	Standard deviation of TLD measurements
Air kerma	RaySafe measurement	B	Dosemeter calibration certificate
ORGAN DOSE ASSESSMENT			
TLD response	Repeatability	A	Repeatability preliminary study
Calibration curve	Angular coefficient	B	Computational software prediction
Calibration curve	Intercept	B	Computational software prediction

The PCXMC 2.0 software version was used for MC calculations. Two approaches were investigated in order to simulate each EOS projection:

(1) A single-shot event, selecting the field size to include the full acquisition range, and entering as input the dose-area-product (DAP) provided by the imaging system (reported in Table 2).
(2) *n* different simulations with contiguous 5 mm high slot beams, each with DAP calculated as DAP_total_/n. *n* was equal to 130 and 210 for the pediatric and adult phantoms, respectively.

In order to evaluate the impact of the beam vertical dimension on the simulation results, for the pediatric patient, calculations were also performed using contiguous 1mm–high slot beams. In the text, we will refer to this computation method as Method (2′).

In all the investigated situations, infinite focus-to-skin distance (FSD) (actually 100 m) was set to avoid beam divergence. Table 4 shows the setup and geometrical simulation parameters. In order to evaluate the impact of the distance, simulations of pediatric examination with Method (1) were performed also at a fixed FSD.

**Table 4:** Setup parameters used for MC simulations. The Labels (1), (2) and (2′), where present, specify the simulation method. For Methods (2) and (2′), the geometric characteristics refer to a single slit beam.

**Parameter**	**Pediatric patient**	**Adult patient**
Phantom data	5 y (height 110 cm; weight 19 kg); no arms in phantom	Adult (height 175; weight 73.5); no arms in phantom
FSD [cm]	10 000 (infinite)	10 000 (infinite)
Beam size [cm]	(1) Width = 44.8; height = 65 (2) Width = 44.8; height = 0.5 (2′) Width = 44.8; height = 0.1	(1) Width = 40 (AP); 32 (LL); height = 105 (2) Width = 40 (AP); 32 (LL); height = 0.5
(Xref; Yref; Zref)	(1) (0; 0; 27.5) (1) (0; 0; −4.75 + 0.5*i) *i* = 0; 129 (2′) (0; 0; −4.95 + 0.1*i) *i* = 0; 650	(1) (0; 0; 43.75) (2) (0; 0; −8.25 + 0.5*i) *i* = 0;209
Projection angle [degree]	AP projection: 270 LL projection: 0	AP projection: 270 LL projection: 0
Cranio-caudal angle [degree]	0	0

Five thousand photons were generated in each simulation. Based on the results of manufacturer annual tests, the X-ray beam quality was simulated with a total filtration of 3.5 mm aluminum (Al) + 0.1 mm copper (Cu). AP and LL projections were simulated separately, entering each time the kVp and DAP value provided by the imaging device. Organ and effective doses were calculated as the sum of the two contributions. For the pediatric patient, Method (2) was applied to simulate acquisitions performed at SL 2, whereas calculations with Method (1) were repeated for each investigated X-ray tube SL, entering each time the corresponding DAP.

## Results

### Comparison of simulation methods

Table 5 shows the results of organ dose and effective dose estimations based on simulation Methods (1) and (2), for both the adult and pediatric patients. The relative difference between the two simulation methods was calculated as

$$ \mathrm{diff}=\frac{\mathrm{D}(1)-\mathrm{D}(2)}{\mathrm{mean}\left(\mathrm{D}(1);\mathrm{D}(2)\right)}. $$

**Table 5:** Organ and effective doses calculated with Methods (1) and (2) for adult and pediatric patients. For each organ, the relative difference between the two simulation approaches is reported.

	**Adult patient**					**Pediatric patient**				
	**Method (1)**		**Method (2)**			**Method (1)**		**Method (2)**		
**Organ**	**Dose (uGy)**	**Error (%)**	**Dose (uGy)**	**Error (%)**	**Diff. %**	**Dose (uGy)**	**Error (%)**	**Dose (uGy)**	**Error (%)**	**Diff. %**
Active bone marrow	107	1	108	0	−1	40	1	41	1	−2
Adrenals	76	15	71	6	7	45	19	41	7	10
Brain	147	2	150	1	−1	56	2	57	1	−2
Breasts	235	3	237	4	−1	84	25	90	8	−7
Colon (large intestine)	152	3	151	1	0	68	4	68	2	−1
Extrathoracic airways	203	10	201	4	1	66	11	68	5	−3
Gall bladder	124	9	128	4	−3	65	8	67	4	−3
Heart	187	3	184	2	2	83	4	83	2	0
Kidneys	78	5	81	2	−3	43	5	44	2	−3
Liver	111	3	110	1	0	51	3	51	1	−2
Lungs	163	2	165	1	−1	73	2	74	1	0
Lymph nodes	164	2	164	0	0	70	2	71	0	−2
Muscle	112	0	112	0	−1	48	1	48	0	−1
Esophagus	106	7	105	5	1	63	11	60	5	4
Oral mucosa	186	6	187	5	−1	71	7	72	4	−2
Pancreas	151	6	147	4	2	72	8	71	4	2
Prostate	135	16	134	5	1	68	33	59	6	14
Salivary glands	199	4	192	4	4	67	7	70	4	−5
Skeleton	199	1	200	0	−1	105	1	107	0	−2
Skin	119	1	120	0	−1	43	2	44	0	−2
Small intestine	134	2	136	1	−1	64	2	65	2	−2
Spleen	206	5	204	3	1	86	6	87	4	−2
Stomach	240	3	239	3	0	99	4	99	3	1
Testicles	206	11	188	5	9	66	31	76	6	−14
Thymus	193	11	203	6	−5	81	7	81	4	0
Thyroid	292	8	305	5	−4	107	14	115	5	−8
Urinary bladder	165	6	167	4	−1	71	9	71	4	0
**Effective dose**	172	1	172	0	0	71	4	73	0	−3

The differences in organ doses ranged from −5 to 9% (average 0%; median −1%) for the adult patient, and from −14 to 14% (average −1%; median −2%) for the pediatric patient. Differences were not statistically significant (95% confidence interval) for any organ.

### The impact of slit dimension

**Figure 3:**
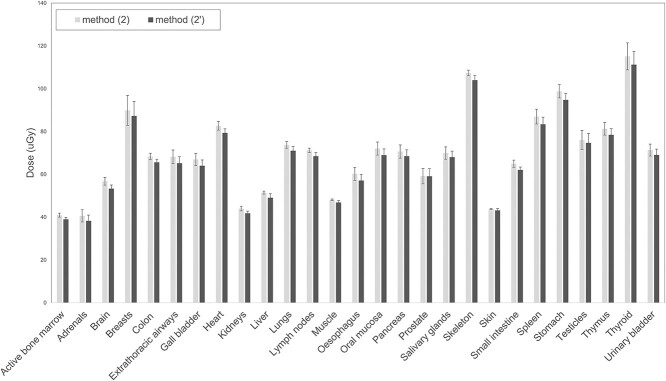
Comparison of organ doses calculated with contiguous 5 mm high (Method (2)) and 1 mm high (Method (2′)) slot beams. Results refer to pediatric acquisitions performed at SL 2. Error bars represent ±2 standard deviations.

Figure 3 compares organ doses calculated using contiguous slot beams of 5 mm (Method (2)) and 1 mm (Method (2′)). Doses calculated with Method (2) were slightly higher than those of Method (2′) with a maximum relative difference of 6% (absolute 4 μGy). Differences were not statistically significant for any organ.

### The impact of FSD

**Figure 4:**
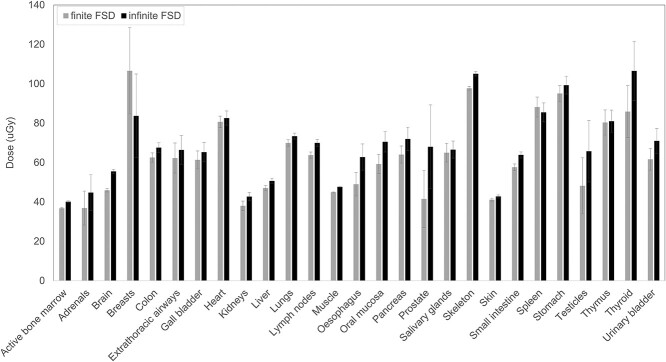
Comparison of organ doses calculated with Method (1) with finite and infinite FSD. Results refer to pediatric acquisitions performed at SL 2. Error bars represent ±2 standard deviations.

Figure 4 compares organ doses calculated with simulation Method (1) for finite and infinite FSD. Doses obtained at infinite FSD were higher than at finite distance for almost all organs, with the exception of breasts. Differences ranged from −27% (for breasts) to +39% (for prostate), with a median value of +7%.

### Comparison of calculated and measured doses

**Figure 5:**
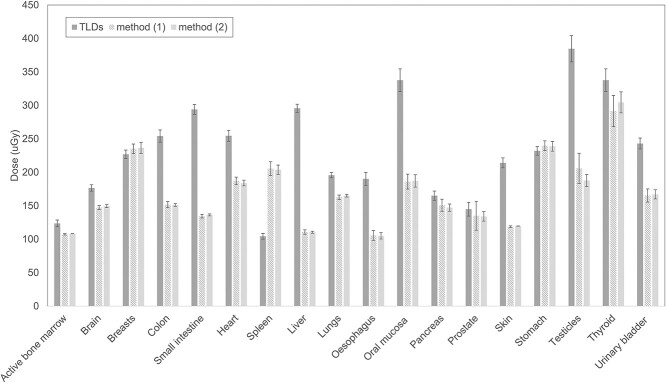
Comparison of organ doses measured with TLDs and computed with PCXMC software for acquisitions performed with the adult phantom. Simulation Methods (1) and (2) are considered. Error bars represent ±2 standard deviations.

**Figure 6:**
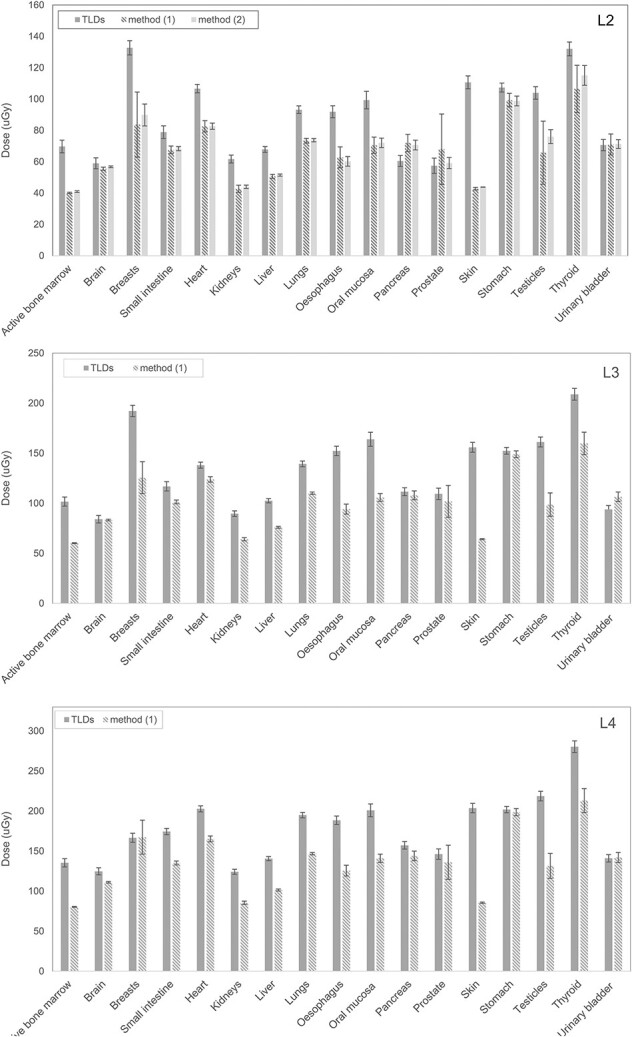
Comparison of organ doses measured with TLDs and computed with PCXMC software for acquisitions performed with the pediatric phantom at SLs 2–4. Where available, simulation Methods (1) and (2) are considered. Error bars represent ±2 standard deviations.

The results of MC simulations and experimental measurements are compared in Figures 5 and 6. For both adult and pediatric patients, doses derived from TLD measurements were higher than the simulated ones for almost all organs. For each organ, the relative difference (Δ) between the measured and calculated doses was computed, as

$$ \Delta =\frac{\mathrm{D}\left(\mathrm{TLD}\right)-\mathrm{D}\left(\mathrm{MC}\right)}{\mathrm{D}\left(\mathrm{TLD}\right)}. $$

For the adult patient Δ ranged from −97 to 63% (average 19%, median 21%) when considering Method (1) and from −95 to 63% (average 19%, median 21%) when considering Method (2). Differences were not statistically significant for 4 out of 18 organs (22%).

For the pediatric patient, Δ ranged from −19% (pancreas) to 61% (active bone marrow) with average values of 20% at SL 2 and 22% at SLs 3 and 4. Differences were not statistically significant for 6 out of 17 organs (35%) for every investigated acquisition velocity.

Effective dose evaluations are shown in Table 6; PCXMC underestimated the effective dose of 26% in the adult and pediatric examinations performed at SL 4, and of 24% in pediatric examinations at SLs 2 and 3.

**Table 6:** Effective dose (μSv) derived from measurements with TLDs and calculated by PCXMC. Error bars represent ±2 standard deviations.

	**Effective dose (μSv)**				**Percentage difference (%)**		
	**TLDs**	**Method (1)**	**Method (2)**	**Method (2′)**	**TLDs versus Method (1)**	**TLDs versus Method (2)**	**TLDs versus Method (2′)**
Adult L4	232 ± 5	172 ± 4	172 ± 1	—	26	26	—
Pediatric L2	94 ± 4	71 ± 3	73 ± 1	70 ± 2	24	22	25
Pediatric L3	140 ± 4	106 ± 4	—	—	24	—	—
Pediatric L4	191 ± 5	141 ± 6	—	—	26	—	—

Based on these results, a good approximation of the effective dose *E* can be derived from the PCXMC-calculated value (${E}_{\mathrm{PCXMC}}$) as $E=1.35\ {E}_{\mathrm{PCXMC}}$. A conservative ±10% uncertainty is recommended.

## Discussion and conclusions

The aim of the work was to evaluate the accuracy of PCXMC for computing organ doses and effective dose in examinations with a slit-beam imaging device. The use of slot-scanning technology allows acquiring true-to-size images of the whole body with a single acquisition, removing the need for digital stitching and avoiding the magnification errors because of the divergent X-ray beam of teleradiography systems. For these reasons, slot-scanning devices—and above all the EOS imaging system—are nowadays widely used for anatomical assessment of the entire musculoskeletal system, especially for the follow-up of chronic disorders such as scoliosis, limb length discrepancy and posture complications. However, no commercial MC software is supplied with specific functions to simulate this X-ray beam geometry. To simulate slot-scanning acquisitions, Kulkarni *et al.*^(20)^ proposed a method using the MC simulation package PENELOPE and penEasy Imaging, whereas Clavel *et al*.^(21)^ developed a GATE model. However, even if not developed for this purpose, PCXMC is still the most widely used software for simulating EOS acquisitions. Nonetheless, to the best of our knowledge, the results have been never formally validated with experimental measurements. Thereby, this study compared the results of PCXMC simulations with those of experimental measurements with physical phantoms and TLDs. For completeness, examinations of both pediatric and adult patients were evaluated and different scanning speeds, corresponding to increasing doses, were investigated. For this purpose, anthropomorphic phantoms representing an adult male and a 5-y-old child were employed.

Two approaches were considered to simulate slot-scanning acquisitions: in Method (1), following the instructions of Hui *et al.*^(15)^, a single simulation with field dimensions to include the full acquisition range was performed; in Method (2), indeed, similarly to Law *et al.*^(14)^, the acquisition range was divided into multiple contiguous 5mm–high slot beams. Organ and effective doses calculated with the two methods were found to be equivalent, both for adult and pediatric patients. The largest discrepancies (14%, absolute 10 μGy) were observed for testicles and prostate in the 5-y-old patient, the organs with the largest uncertainties (31 and 33%, respectively). Relative differences in effective doses were equal to 0 and 3% (absolute 2 μGy) for adult and pediatric patients, respectively.

In order to evaluate the impact of the slit width on the simulations, calculations performed with Method (2) were replicated using 1mm–high beams. A maximum relative difference of 6% was recorded. Differences were not statistically significant for any organ.

To better simulate the acquisition setup and avoid beam divergence, an infinite FSD was set. This choice provided organ doses on median 7% higher than for finite FSD. In 18 out of 27 (67%) of the cases, differences were not statistically significant (95% confidence interval). Statistically significant differences were found especially for large organs (active bone marrow, lymph nodes, muscles, skeleton and small intestine), and for organs near the border of the beam (brain and oral mucosa), which are expected to be more influenced by the effect of beam divergence.

Organ doses derived from TLD measurements were generally higher than the simulated doses, for both pediatric and adult patients. Statistically significant discrepancies were observed for almost all organs, highlighting that PCXMC is not suited for simulating a continuous scanning irradiation. However, interestingly, a similar relative difference between TLD measurements and simulations was recorded in effective dose assessments: regardless of acquisition SL and simulation method, PCXMC underestimated effective dose, with respect to the measured value, of about 25% for both the pediatric and adult patient.

The results were compared with those of other studies in the literature; good agreement was observed with doses measured by Damet *et al.*^(22)^, who performed similar TLD measurements with a pediatric phantom. A very good agreement was achieved also with the results of the recent work of Pedersen *et al.*^(23)^, who performed TLD in-phantom measurements at SL 4. Branchini *et al*.^(24)^ measured considerably higher doses for all the investigated organs, but results were not comparable as their measurements were performed at SL7. Hamzian *et al*.^(25)^ performed a MC simulation with PCXMC for a whole-body examination at SL 4 and found considerably lower doses than those measured with TLDs in this study. However, a good agreement was observed with the results of the MC simulation.

Based on these results, the use of PCXMC for organ dose evaluations in exams with slot-scanning devices is discouraged. However, the software is a useful tool to estimate the effective dose in whole-body EOS examinations, by multiplying the calculated value by a 1.35 correction factor. The same factor applies for examinations of patients of different ages, and for every scanning SL. For the effective dose calculation, a single simulation with infinite FSD and field dimensions to include the full acquisition range is the best approximation and the least time-consuming choice.

## Data Availability

The original contributions presented in the study are included in the article, further inquiries can be directed to the corresponding author.
